# Seroprevalence and Molecular Epidemiology of *Leptospira* spp. Infecting Dogs in the Yangtze River Region of China

**DOI:** 10.1155/tbed/5728490

**Published:** 2025-12-03

**Authors:** Yue Ding, Shilei Zhang, Wenlong Zhang, Sheng Sun, Xufeng Xie, Yongguo Cao

**Affiliations:** ^1^State Key Laboratory for Diagnosis and Treatment of Severe Zoonotic Infectious Diseases, Key Laboratory for Zoonosis Research of the Ministry of Education, College of Veterinary Medicine, Jilin University, Changchun 130062, Jilin, China; ^2^Department of Clinical Veterinary Medicine, College of Veterinary Medicine, Jilin University, Changchun 130062, Jilin, China; ^3^State Key Laboratory of Pathogen and Biosecurity, Changchun Veterinary Research Institute, Chinese Academy of Agricultural Sciences, Changchun, Jilin, China

**Keywords:** dogs, isolated strain, leptospirosis, MAT, MLST, serogroup, WGS, Yangtze River region

## Abstract

Leptospirosis, a globally re-emerging zoonosis caused by pathogenic *Leptospira* species, poses escalating public health challenges in rapidly urbanizing regions. Canines, as significant reservoir hosts, are increasingly regarded as effective sentinels for human leptospirosis risk. This study assessed the seroprevalence of pathogenic *Leptospira* in dogs across multiple provinces and regions along the Yangtze River in China. From 2021 to 2023, a total of 1517 canine serum samples were collected from Shanghai, Jiangsu, Anhui, Jiangxi, Hunan, Hubei, Chongqing, Sichuan, and Yunnan. In addition, a tissue sample was obtained from an infected dog, leading to the successful isolation and culture of one *Leptospira* strain. Microscopic agglutination test (MAT) results indicated an overall seroprevalence of 46.41% (704/1517), predominantly involving *L. interrogans* serogroups Canicola (72.73%, 512/704) and Icterohaemorrhagiae (28.68%, 202/704), followed by Ballum (18.04%, 127/704) and Australis (17.90%, 126/704). Organ examination and histopathological analysis identified severe pulmonary hemorrhage induced by the isolated strain as the primary cause of canine mortality. Whole-genome sequencing (WGS) and multilocus sequence typing (MLST) based on seven housekeeping genes classified the isolate as *L. interrogans* serovar Australis, sequence type (ST) ST93. These findings reveal a high seroprevalence of pathogenic *Leptospira* in dogs within the Yangtze River region, consistent with the distribution of locally prevalent serogroups, and underscore the potential public health risk posed by this zoonotic pathogen in the area.

## 1. Introduction

Leptospirosis is a global zoonotic disease caused by Gram-negative bacteria in the genus *Leptospira*. Pathogenic *Leptospira* infection causes life-threatening conditions such as leptospiral pulmonary hemorrhage syndrome and Weil's disease [1, 2], which cause more than 1 million cases and 58,900 deaths per year [3]. The genus *Leptospira* comprises highly diverse bacteria, including at least 69 pathogenic species, with over 300 serovars [4–6]. To date, at least 200 species of animals have been identified as natural carriers of pathogenic *Leptospira* strains worldwide [7, 8]. Although leptospirosis incidence and mortality have decreased nationwide in recent years, local rates are still above the average [9]. Recently, increasing reports of canine leptospirosis have demonstrated that the canine is an important *Leptospira*-carrying animal host [10–14]. In contrast to other pets, dogs are more susceptible to infection due to exposure to pathogenic *Leptospira* in the environment [15]. Dogs have a significant impact on the transmission of leptospirosis to humans, as well as on the recirculation of the pathogen among both humans and animals [15, 16]. However, there is limited research on the epidemiology and serogroups of pathogenic *Leptospira* in dogs in the Yangtze River Basin of China.

Benefit to the continuous improvement of the public healthcare system in China, the average incidence rate of human leptospirosis has decreased from 0.32 per 100,000 in 2000 to 0.07 per 100,000 in 2022 [17]. However, this decline has indirectly contributed to the ongoing neglect of leptospirosis. Abundant water systems and frequent natural disasters in the Yangtze River region, facilitate the dissemination and transmission of pathogens [1, 18, 19]. In addition to the close relationship between the hydrological system and climate, the regions along the Yangtze River are characterized by active water-related activities, both recreational and occupational [17, 20]. These environmental factors collectively create favorable conditions for the persistent presence of *Leptospira*. In regions along the Yangtze River, such as Jiangxi [21], Anhui [22], Hubei [23, 24], Chongqing [25], and Sichuan [20], there continue to be reports of cases, detection, and pathogen isolation of leptospirosis. The objective of this investigation was to ascertain the serogroups of pathogenic *Leptospira* species in dogs in the Yangtze River region to gain a comprehensive understanding of local *Leptospira* epidemiology, which could be the basis for improving preventive strategies.

## 2. Materials and Methods

### 2.1. Study Area

**Figure 1:**
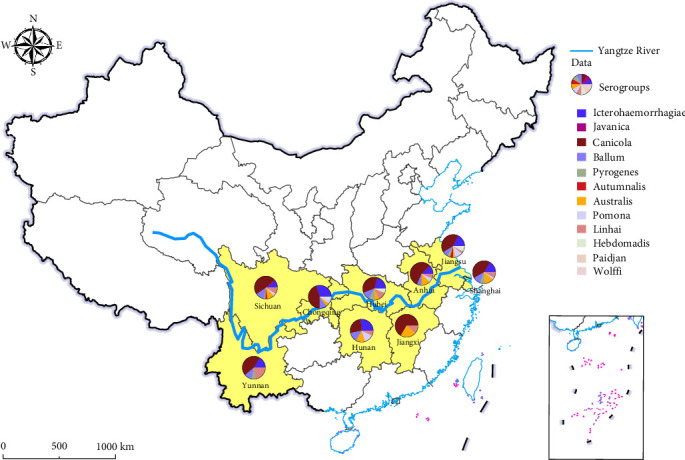
Distribution of sampling areas and proportion of positive serological results.

The research was carried out in the Yangtze River Basin of China, which includes Shanghai, Jiangsu, Anhui, Hunan, Hubei, Jiangxi, Chongqing, Sichuan, and Yunnan (Figure 1). This region of the Yangtze River has a subtropical monsoon climate, which is humid and rainy all year, with a long rainy season.

The samples were randomly collected from veterinary clinics located along the Yangtze River region. We collected blood samples randomly from January 2021 to December 2023. The data obtained from canine blood encompasses various factors, including breed, age, sex, and the timing of blood collection.

### 2.2. Sample Preparation

A total of 1517 blood samples were obtained from the saphenous vein of dogs using blood lancets and EDTA tubes (YA1293, Solarbio Science). Portions of whole blood samples (200 *μ*L per sample) were stored at −20°C for PCR detection. The remaining blood samples were incubated at 37°C for 30 min, followed by centrifugation at 3000 rpm for 5 min. The separated serum was then stored at −20°C for microscopic agglutination test (MAT) detection.

### 2.3. MAT

We tested all the samples against the serogroups of the standard strain (Table S1). The titer of the antisera was determined using the MAT [26]. In this test, 15 pathogenic *Leptospira* serogroups and Patoc were used as antigens, with a concentration of 1 × 10^8^ cells/mL for *Leptospira*. As previously mentioned [27, 28], Samples were stored at −20°C until testing. Serum was heat-inactivated at 56°C for 30 min. Serial twofold dilutions starting at 1:100 were mixed with an equal volume of *Leptospira* in a microtiter plate. After 2 h incubation at 30°C, agglutination was assessed via dark field microscopy. The final titer was defined as the reciprocal of the highest dilution showing ≥50% agglutination compared to the negative control; a titer above 100 was considered positive.

### 2.4. Blood Biochemistry and Histopathology

During the sampling period, a 5-year-old female East German Shepherd was identified with clinical signs, including fever, lethargy, and icterus, of the conjunctiva. Based on these symptoms, a presumptive diagnosis of leptospirosis was made. Blood samples were collected via venipuncture for biochemical analysis. The dog was subsequently administered antibiotic therapy combined with intravenous fluid support. Despite medical intervention, the animal succumbed to acute illness 2 days after initial presentation. Blood biochemistry was measured using an SMT-120VP (Seamaty, China). The infected animal succumbed on day 3 postdiagnosis. With the owner's consent, a necropsy was conducted for pathological examination with tissue collection. Liver, kidney, lung, and splenic tissues from hamsters were collected, fixed in 10% neutral buffered formalin, and processed into paraffin sections stained with H&E. Slides were scanned with a Pannoramic MIDI digital system (3DHISTECH). As previously described [29], tubulointerstitial nephritis, pulmonary, hepatic/splenic inflammation, based on inflammatory foci per 10 × 10 field.

### 2.5. *Leptospira* Culture and Isolate

*Leptospira* strains were obtained from infected dog urine collected via sterile puncture to avoid contamination. The samples were inoculated into five tubes of Ellinghausen–McCullough–Johnson–Harris (EMJH) liquid medium and transferred to the laboratory. Cultures were maintained at 29°C and examined weekly under dark field microscopy for Leptospira growth over 12 weeks [30, 31].

### 2.6. Whole Genome Sequencing (WGS) and Pangenome Analysis

The WGS of the Leptospira isolate was conducted at Sangon Biotech (Shanghai, China). The whole genome DNA was randomly fragmented to an average size of 200–400 bp. The selected fragments were through end-repair, 3′ adenylated, adapters-ligation, PCR amplifying. After purification with the magnetic beads, the library was qualified by the Qubit 4.0 fluorometer, and the length of the library was assessed by the 2% agarose gel electrophoresis. The qualified libraries were sequenced on the MGI DNBSEQ-T7. After sequencing, raw reads were filtered via Fastp (v 0.23.0) by removing adaptors and low-quality reads, then clean reads were obtained. Genome survey was performed using Jellyfish (v 2.3.0) for k-mer counting and GenomeScope2 for genome size and heterozygosity estimation based on clean reads. Genome assembly was done using SPAdes (v 3.5.0), and the Gapfiller (v 1.11) was used for filling gaps. In addition, Pilon (v 3.5.0) improves draft genomes accuracy by correcting base errors and filling gaps. Sequencing data have been uploaded to NCBI BioProject, reference PRJNA1308474.

For pangenome analysis, Roary (v 3.13.0) processed all protein sequences through BLASTp clustering, defining orthologous clusters where: core genes represented clusters present in all strains (90% ≤ strains ≤ 100%), dispensable genes comprised variably present clusters across subsets of strains (15% ≤ strains < 89%), and strain-specific genes denoted clusters exclusive to individual strains (0% ≤ strains < 15%).

### 2.7. Multilocus Sequence Typing (MLST)

DNA from isolated *Leptospira* was extracted using the commercial TIAN-amp Bacteria DNA kit following the manufacturer's instructions (TIANGEN, China). MLST #1 employs seven housekeeping genes (glmU, pntA, sucA, caiB, tpiA, pfkB, and mreA) for analysis. Specific primers for the housekeeping gene and the PCR program used were previously described (Table S2) [32]. Bidirectional sequencing was conducted by Sangon Biotech (Shanghai, China). The specified gene (adk, icdA, lipL32, lipL41, rrs2, secY) sequences for MLST schemes #2 and #3 were extracted from the WGS data. Subsequently, the corresponding loci of these sequences were identified using the Batch Sequence Query tool in the Leptospira database on PubMLST. Finally, the sequence types (STs) of the isolates for MLST schemes #1, #2, and #3 were determined based on their allelic profiles.

### 2.8. Phylogenetic Analysis

Core genome MLST (cgMLST)-based phylogenetic trees were constructed by importing allele data from the Public databases for molecular typing and microbial genome diversity (Pubmlst) [33] into SeqSphere + (Ridom). Subsequently, a neighbor-joining tree (NJT) was generated based on the allele differences among the isolate and reference strain.

### 2.9. Data Analysis

Results with *p*-values < 0.05 were considered to indicate statistical significance. The chi-square test (*χ*^2^) was used to measure the differences in proportions between generated categories, including breed, age, sex, and the time of sample collected.

## 3. Results

### 3.1. Region and Positive Serogroup Results Analyze

To determine the seroprevalence of pathogenic Leptospira infection in dogs along the Yangtze River region, serum samples were tested using the MAT. The serological test results of a total of 1517 samples indicated an overall seropositivity rate of 46.41% (704 out of 1517). The primary positive serogroups identified were *L. interrogans* serogroups Canicola at 72.73% (512/704), Icterohaemorrhagiae at 28.69% (202/704), Ballum at 18.04% (127/704), Australis at 17.9% (126/704), Grippotyphosa at 12.78% (90/704), Pyrogenes at 10.23% (72/704), Pomona at 7.53% (53/704), Autumnalis at 2.84% (20/704), Sejroe at 1.42% (10/704), Javanica at 0.99% (7/704), and Bataviae at 0.28% (2/704) (Table 1). Analysis of regional factors revealed that all provinces exhibited high seropositivity rates and complex epidemic serogroups, indicating a widespread and diverse distribution of *Leptospira* (Figure 1).

**Figure 2:**
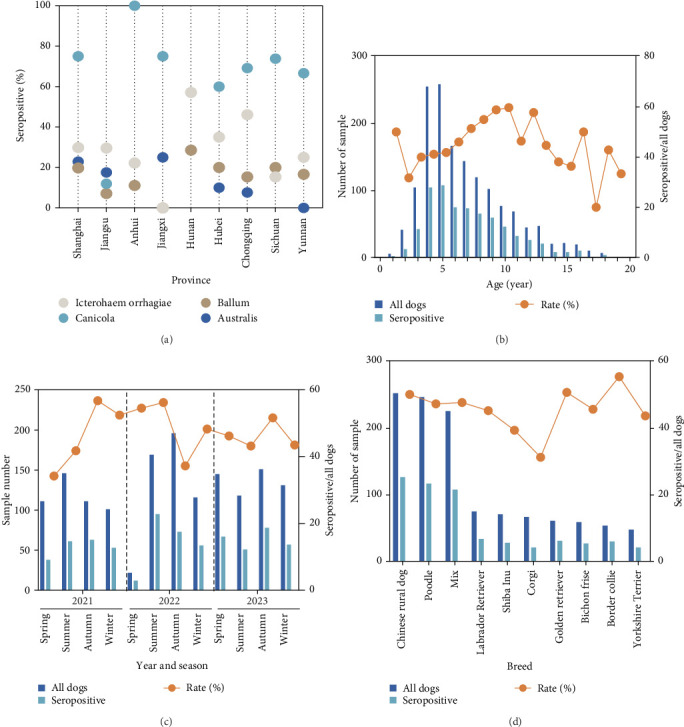
Further analysis was conducted on sampling time, age, predominant regional serogroups, and breed factors. (A) The correlation between provinces and the predominant circulating serogroups. (B) The relationship between age and seropositive samples. (C) The association between sample collection time and the season of seropositive samples. (D) The correlation between breed and seropositive samples.

Analysis of various factors, including gender, breed, age, sampling year, and sampling season. The results indicated that these factors did not have a significant effect on the seropositivity rate (Table 2). Further analysis of regional distribution and predominant serogroups revealed that all provinces detected the *L. interrogans* serogroup Icterohaemorrhagiae. However, the *L. interrogans* serogroup Australis was not found in Anhui, Hunan, and Sichuan provinces; the Ballum serogroup was absent in Jiangxi province; and the Canicola serogroup was not detected in Hunan province. These findings suggest that certain serogroups exhibit regional limitations (Figure 2A). The analysis of age in seropositive dogs showed that the seropositivity rate steadily increased from 2 to 10 years of age (Figure 2B). This trend may be related to the cumulative exposure to outdoor activities over time, indicating a widespread presence of potentially pathogenic *Leptospira* in the environment. Analysis by sampling periods indicates that samples collected in summer and autumn showed higher risk levels compared to those collected in spring and winter (Figure 2C). The analysis of positive samples among dog breeds indicated that the main positive breeds were Chinese rural, Poodle, and mixed-breed dogs. Although the sample sizes for Golden Retrievers and Border Collies were smaller, these breeds showed a comparatively higher rate of positivity (Figure 2D). These results suggest that the transmission and pathogenic risk of *Leptospira* in the areas along the Yangtze River are relatively high.

### 3.2. Pathological Analysis and Pathogen Isolated

Upon identification of the suspected diseased dogs, blood biochemistry was promptly conducted. Significant elevations were observed in alkaline phosphatase (ALP), alanine aminotransferase (ALT), total bilirubin (TBIL), and blood urea nitrogen (BUN) levels, indicating severe liver and kidney damage. As the disease had progressed to an advanced stage, 2 days of treatment failed to slow its progression. Blood biochemistry indicators during the terminal phase revealed the development of liver and kidney failure (Table S3).

**Figure 3:**
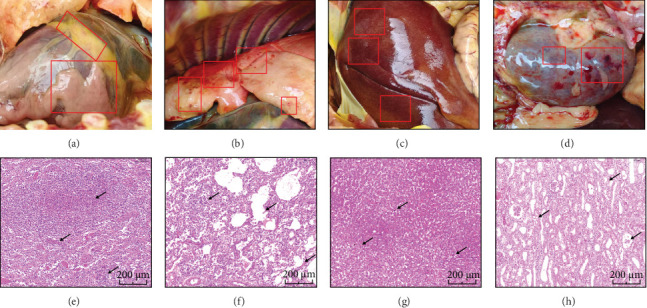
Anatomical diagrams and histopathology of Canine *Leptospira* Infection Pathological images of the case dog, (A) the heart. (B) the lungs. (C) the liver and (D) the kidneys. Histopathological images of the case dog, (E) the spleen. (F) the lungs. (G) the liver and (H) the kidneys. The scale bar is 200 *μ*m.

With the owner's consent, we promptly conducted a pathological autopsy and histopathological examination on the diseased dog. The autopsy findings revealed suspected inflammatory exudation in the myocardium, multiple hemorrhagic spots in the lungs, possible hepatic congestion or early cell degeneration in the liver, and numerous hemorrhagic areas in the kidneys, indicating severe liver and kidney failure accompanied by myocardial inflammation (Figure 3A–D). Histopathological analysis revealed extensive infiltration of inflammatory cells within the spleen, accompanied by widening of the splenic cords in the red pulp and disruption of normal tissue architecture. In the lungs, alveolar structures were disorganized, with thickened septa and inflammatory cell infiltration in the interstitial regions. The liver exhibited widespread inflammatory cell infiltration along with interstitial nephritis. In the kidney, inflammatory cells infiltrated the interstitial area, renal tubules showed abnormal morphology and epithelial cell degeneration, and the glomerular structures appeared unclear with increased cellularity (Figure 3E–H).

### 3.3. Pangenome and Phylogenetic Analysis

**Figure 4:**
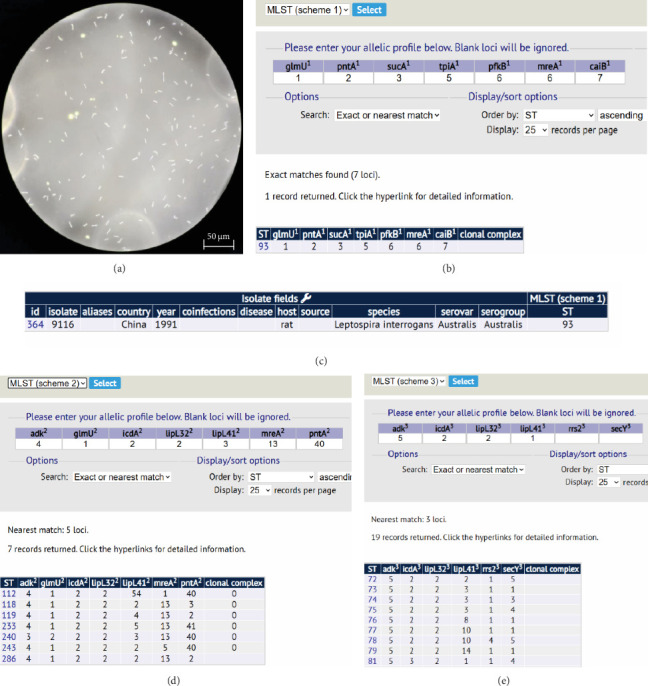
Dark field microscopic examination and MLST analysis of *Leptospira* isolates. (A) The leptospiral isolate was observed under dark field microscopy at a magnification of 400× following cultivation. (B) Multilocus sequence typing (MLST) analysis based on seven housekeeping genes revealed a match with MLST profile #1 in the PUBMLST database. (C) Sequence type ST93 was identified as corresponding to isolate ID 364 (strain no. 9116) in the PUBMLST database. (D) A match with MLST profile #2 was determined through analysis of seven housekeeping genes in the PUBMLST database. (E) Similarly, MLST profile #3 was assigned based on the matching of seven housekeeping gene sequences within the PUBMLST database.

**Figure 5:**
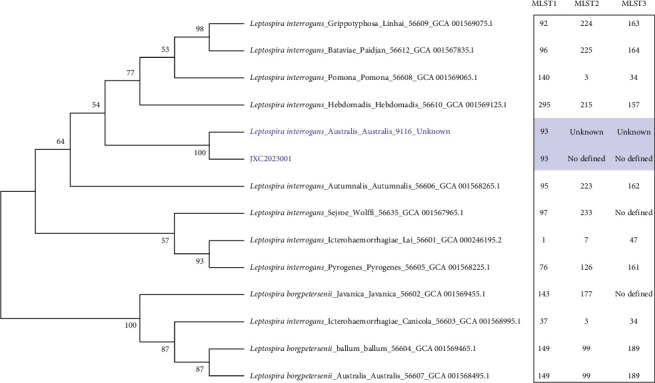
Neighbor-joining tree based on the allele differences among the isolate and reference strain.

**Figure 6:**
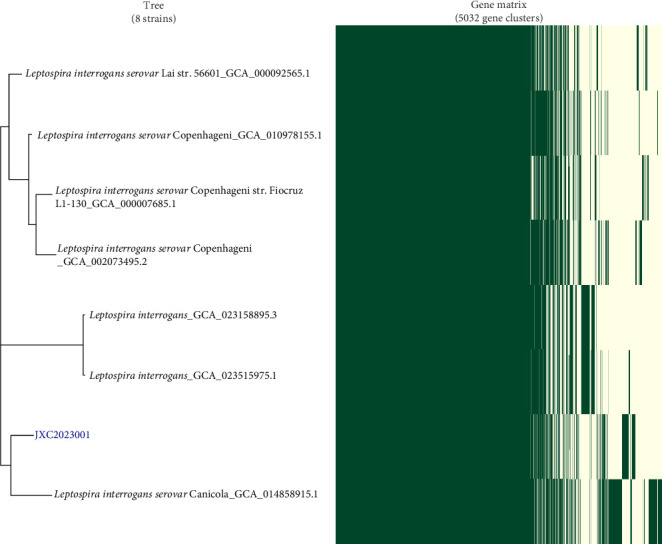
Comparative pangenome analysis of *Leptospira* isolates.

The urine sample was subjected to continuous subculturing in EMJH liquid medium, resulting in the isolation of a *Leptospira* strain JXC2023001 (Figure 4A). WGS and MLST scheme #1 were successfully performed, identifying the isolate as ST 93. This classification places the isolate within the *L. interrogans* serogroup Australis, serovar Australis (Figure 4B,C). However, MLST analyses #2 and #3 did not yield matching ST classifications (Figure 4D,E). An allele difference NJT was constructed based on the isolates and reference strains (Figure 5). A total of 12 distinct reference strain STs were employed (Table S1). The Illumina WGS method produced over 4.6 million base pairs and had a median GC content of 35.4%. Pan-genome analysis revealed 2994 core genes, 1218 shell genes, and 820 cloud genes among the JXC2023001 *Leptospira* isolates. Then, a hierarchical clustering dendrogram was generated based on the presence/absence of pan-genome gene clusters (Figure 6).

## 4. Discussion

In China, leptospirosis represents a prevalent and widely distributed zoonotic disease [34, 35]. As one of the primary companion animals in the country, dogs have increasingly been recognized as valuable sentinels for a number of zoonotic pathogens [16, 36]. In this study, a serological investigation was conducted to assess the seroprevalence and identify the predominant pathogenic *Leptospira* serogroups in dogs across the Yangtze River region between 2021 and 2023. The average annual seropositivity rate throughout this period was 46.41%. Findings reveal that the most frequently detected serogroups in the region were *L. interrogans* serogroups Canicola (72.73%), Icterohaemorrhagiae (28.69%), Ballum (18.04%), and Australis (17.90%).

The prevalence rate obtained from our findings was significantly higher than the reported in studies performed in other countries (25.10% in the Los Rios Region, Chile [37], 19.7% in Brazil [38], and 21.3% in Temuco, Chile [39]). A study conducted in 2007 revealed that the most common serogroups among the 868 patients in the three Gorges area (Chongqing section) were *L. interrogans* serogroups Icterohaemorrhagiae (6.45%), Australis (6.11%), Hebdomadis (6.68%), and Autumnalis (3.46%) [40]. Our study results indicate that the predominant serogroups circulating among dogs in Jiangxi Province are the *L. interrogan* serogroups Canicola (50.00%) and Australis (16.67%). In contrast, previous research conducted from 2002 to 2015 on rodents in Jiangxi Province identified the primary circulating serogroups as *L. interrogan* serogroups Icterohaemorrhagiae (61.10%), Javanica (17.20%), and Australis (9.73%) [21]. This discrepancy may be attributed to the differences in host species targeted for screening. Therefore, our research indicates that there may have been a shift in the seroprevalence in the Yangtze River region over the past decade.

Based on the present findings, the highest seropositivity rate for *L. interrogans* serogroup Canicola was detected in regions along the Yangtze River, whereas serogroup Icterohaemorrhagiae demonstrated the broadest geographical distribution. Furthermore, leptospirosis risk levels exhibited a discernible association with the summer and autumn seasons, as well as with animal age. This seasonal variation may be attributable to elevated precipitation levels and increased frequency of outdoor activities during these periods. In alignment with prior research, Wang et al. [17], in their analysis of leptospirosis epidemiological trends in China from 1955 to 2022, identified tropical and subtropical regions of southern China as endemic high-risk zones. Their study indicated that human leptospirosis incidence was predominantly correlated with annual average precipitation, with outbreak peaks occurring in summer and autumn, most notably in August.

During molecular characterization of the isolates, MLST scheme #1 classified the strain as ST ST93. Although conserved alleles corresponding to schemes #2 and #3 were identified from WGS data, no corresponding matches were identified in the PubMLST database (Figure 4D,E). According to current records in PubMLST, ST93 was first isolated in 1991 in Sichuan, China, and is exclusively annotated under MLST scheme #1. This limited scheme coverage likely accounts for the inability to assign the strain JXC2023001 to defined types under schemes #2 and #3.

This study has several limitations that should be acknowledged. First, information regarding vaccination history was not collected during the sampling process. In China, commercially available canine leptospirosis vaccines—such as Zoetis's Vanguard Plus 5-CVL and Intervet's PetBio *Leptospira* bivalent vaccine (*Leptospira Canicola–Icterohaemorrhagiae* Bacterin)—are widely used. As a result, the observed seroprevalence of *L. interrogans* serogroups Icterohaemorrhagiae and Canicola is likely influenced by vaccine-induced antibodies, though no positive cases were detected in certain regions. Nevertheless, this factor does not undermine the overall conclusions of the study. The potential distribution and risk associated with *L. interrogans* serogroup Icterohaemorrhagiae remain considerable in areas along the Yangtze River. Second, the limited sample size, along with insufficient regional coverage in some areas, may constrain the generalizability of local conditions. Accordingly, the analysis primarily focused on several major circulating serogroups.

Previous research on leptospirosis in rodents has predominantly been conducted in regions characterized by low urbanization. In contrast, the present study reveals that densely urbanized environments also present a non-negligible risk of leptospirosis transmission. Consequently, future investigations should consider the use of domestic dogs as sentinel species for human leptospirosis, given their potential to offer more representative epidemiological indicators than rodents. Furthermore, recent climatic shifts in central China—such as the prolongation of the rainy season and the northward migration of precipitation belts—may facilitate alterations in the geographic distribution of leptospirosis risk. Thus, enhanced vigilance and systematic surveillance are recommended in central, northern, and northwestern China. The identification of circulating serogroups through serological and molecular assays could inform the development of targeted canine vaccines, which may contribute to leptospirosis prevention. Strengthening monitoring systems in the future would support more comprehensive disease surveillance.

## Figures and Tables

**Table 1:** Analysis of positive samples by prevalent serogroups and provinces.

Serogroup/province	Shanghai	Jiangsu	Anhui	Jiangxi	Hunan	Hubei	Chongqing	Sichuan	Yunnan	Total^a^	Serogroup Seroprevalence (^a^/704) (%)
Icterohaemorrhagiae	133	37	2	0	4	7	6	10	3	202	28.69
Javanica	5	0	0	0	0	0	0	2	0	7	0.99
Canicola	334	82	9	6	4	12	9	48	8	512	72.73
Ballum	88	15	1	0	2	4	2	13	2	127	18.04
Pyrogenes	57	9	0	0	0	3	0	2	1	72	10.23
Autumnalis	8	5	0	0	0	0	0	3	0	20	2.84
Australis	102	22	2	2	2	2	1	10	0	126	17.9
Pomona	23	7	1	0	2	0	1	4	0	53	7.53
Grippotyphosa	63	16	1	1	1	4	0	9	4	90	12.78
Hebdomadis	2	0	0	0	0	2	1	0	0	10	1.42
Bataviae	2	1	0	0	0	0	0	0	0	2	0.28
Sejroe	0	0	0	0	0	0	0	0	0	0	0.00
Seropositive^b^	445	125	9	8	7	20	13	65	12	704	—
All dogs^c^	950	260	13	12	13	51	24	164	30	1517	—
Province Seroprevalence (^b^/^c^) (%)	46.84	48.08	69.23	66.67	53.85	39.22	54.17	39.63	40.00	46.41	—

^a^Total number of samples within the respective serogroups.

^b^Total number of seropositive samples within the respective province.

^c^Total number of samples within the respective province.

**Table 2:** Seropositivity rate and multivariate analysis.

Variables		All dogs, *n* = 1517	Seropositive, *n* = 704	Seroprevalence (%)	*p*-Value
Gender	Male	732	340	46.45	*p* > 0.05
	Female	785	364	46.37	

Breed	Breed	1038	469	45.18	*p* > 0.05
	Mix	226	108	47.79	
	Native	253	127	50.20	

Age (year)	1–7	974	421	43.22	*p* > 0.05
	＞7	543	283	52.12	

Year	2021	469	215	45.84	*p* > 0.05
	2022	503	236	46.92	
	2023	545	253	46.42	

Season	Spring	278	117	42.09	*p* > 0.05
	Summer	433	207	47.81	
	Autumn	458	214	46.72	
	Winter	348	166	47.70	

## Data Availability

The data that support the findings of this study are available in the supporting information of this article.

## Supporting Information

Supporting Information Table S1. Leptospira strains used in this study. Table S2. Information of Loci Proposed for MLST. Table S3. Blood biochemistry of suspected diseased dogs.
