# Supplementary material for: Seroprevalence and Molecular Epidemiology of Leptospira spp. Infecting Dogs in the Yangtze River Region of China
Source: Transbound Emerg Dis. 2025 Dec 3;2025:5728490. doi: 10.1155/tbed/5728490 (PMC12695420; doi:10.1155/tbed/5728490)
Supplement: Supporting Information — Table S1. Leptospira strains used in this study. Table S2. Information of Loci Proposed for MLST. Table S3. Blood biochemistry of suspected diseased dogs. [file 5728490.f1.docx]

**Table S1** *Leptospira* strains used in this study.

| Serotype and strain | Species | Status | Assembly accession |
| --- | --- | --- | --- |
|  |  |  |  |
| Lai 56601 | *L. interrogans* | Pathogenic | GCA_000246195.2 |
| Javanica 56602 | *L. borgpetersenii* | Pathogenic | GCA_001569455.1 |
| Canicola 56603 | *L. interrogans* | Pathogenic | GCA_001568995.1 |
| Ballum 56604 | *L. borgpetersenii* | Pathogenic | GCA_001569465.1 |
| Pyrogenes 56605 | *L. interrogans* | Pathogenic | GCA_001568225.1 |
| Autumnalis 56606 | *L. interrogans* | Pathogenic | GCA_001568265.1 |
| Australis 56607 | *L. borgpetersenii* | Pathogenic | GCA_001568495.1 |
| Pomona 56608 | *L. interrogans* | Pathogenic | GCA_001569065.1 |
| Linhai 56609 | *L. interrogans* | Pathogenic | GCA_001569075.1 |
| Hebdomadis 56610 | *L. interrogans* | Pathogenic | GCA_001569125.1 |
| Paidjan 56612 | *L. interrogans* | Pathogenic | GCA_001567835.1 |
| Wulffi 56635 | *L. interrogans* | Pathogenic | GCA_001567965.1 |

**Table S2** Information of Loci Proposed for MLST.

| Gene | Primer 5' to 3' | Annealing temperature（℃） | Size of PCR Product (bp) |
| --- | --- | --- | --- |
| GlmU | GGAAGGGCACCCGTATGAA  TCCCTGAGCGTTTTGATTT | 50 | 557 |
| PntA | TGCCGATCCTACAACATTA  AAGAAGCAAGATCCACAACTAC | 52 | 638 |
| SucA | AGAAGAGGCCGGTTATCATCAG  CTTCCGGGTCGTCTCCATTTA | 52 | 560 |
| TpiA | AAGCCGTTTTCCTAGCACATTC  AGGCGCCTACAAAAAGACCAGA | 52 | 534 |
| PfkB | CCGAAGATAAGGGGCATACC  CAAGCTAAAACCGTGAGTGATT | 52 | 560 |
| MreA | AAAGCGGCCAACCTAACACC  CGATCCCAGACGCAAGTAAG | 52 | 602 |
| CaiB | CAACTTGCGGAYATAGGAGGAG  ATTATGTTCCCCGTGAYTCG | 46 | 650 |

**Table S3** Blood biochemistry of suspected diseased dogs.

| Item^*^ | 7.25 (Symptoms appear) | 7.27 (On verge death) | Normal range |
| --- | --- | --- | --- |
| ALB | 2.7 | 3.0 | 2.6-4.6 |
| TP | 6.1 | 4.4 | 5.2-8.2 |
| GLU | 91 | 276 | 60-132 |
| ALP | 759 | 422 | 0-212 |
| ALT | 161 | 470 | 0-88 |
| GGT | <10 | <10 | 0-10 |
| TBIL | 4.4 | 16.8 | 0-0.9 |
| AMY | 535 | 1532 | 400-1500 |
| LIPA | 63 | >300 | 0-125 |
| BUN | 58.6 | 208 | 6-26 |
| CREA | 2.81 | 11.34 | 0.4-1.6 |
| CA | 11.5 | 12.8 | 7.9-12 |
| PHOS | 9.3 | 25.4 | 2.5-6.8 |
| GLOB | 3.4 | 1.4 | 2.2-4.6 |

^*^ALB (Albumin), TP (Total protein), GLU (Glucose), ALP (Alkaline phosphatase), ALT (Alanine aminotransferase), GGT (γ-glutamyl transpeptidase), TBIL (Total bilirubin), AMY (Amylase), LIPA (Lysosomal acid lipase), BUN (Blood urea nitrogen), CREA (Creatinine), CA (Calcium), PHOS (Phosphorus), GLOB (globulin).
